# Therapeutic Potential of Nanoparticle-loaded Hydroxyurea on Proliferation of Human Breast Adenocarcinoma Cell Line

**DOI:** 10.22037/ijpr.2020.1100921

**Published:** 2020

**Authors:** Fateme Azemati, Bahman Jalali Kondori, Hadi Esmaeili Gouvarchin Ghaleh

**Affiliations:** a *Department of Biology, School of Basic Sciences, Science and Research Branch, Islamic Azad University, Tehran, Iran. *; b *Department of Anatomical Sciences, Faculty of Medicine, Baqiyatallah University of Medical Sciences, Tehran, Iran. *; c *Baqiyatallah Research Center for Gastroenterology and Liver Diseases (BRCGL), Baqiyatallah University of Medical Sciences, Tehran, Iran. *; d *Applied Virology Research Center, Baqiyatallah University of Medical Sciences, Tehran, Iran.*

**Keywords:** Hydroxyurea, Fe_3_O_4_ NP, Polyethylene glycol, MCF-7 Cells, Cytotoxicity, Radiation, Hyperthermia

## Abstract

Although Hydroxyurea is one of the most widely used drugs in treating breast cancer, the use of it leads to some side effects. Hence, in order to reduce complications of treatment and increase its efficiency, drug delivery has been attracted more attention. Present study included three stages. The first stage was involved in the synthesis of nanoparticles-loaded Hydroxyurea that its characteristics were evaluated by using scanning electron microscopy and Zetasizer system. In the second stage, cultured MCF-7 cells were undergone treatments by Hydroxyurea and Nanoparticles-loaded Hydroxyurea in various concentrations. In the third stage, the MCF-7 was treated by IC_50_ of Hydroxyurea and nanoparticles-loaded Hydroxyurea which are in combination with radiation and hyperthermia. Afterward, the viable of cell, apoptosis, and levels of caspase-8 and-9 proteins were assessed. The average size and the potential surface of nanoparticles and nanoparticles-loaded Hydroxyurea were 26 nm, 48 nm, 3.86 mV, and -29.3 mV, respectively. Results of MTT assay and apoptosis represented that the percentage of cytotoxicity in the treated groups by in combination group and nanoparticles-loaded Hydroxyurea was significantly increased in comparison with Hydroxyurea. This increase was dependent on the concentration of nanoparticles-loaded Hydroxyurea. Nevertheless, the activity of caspase-8 shows any significant changes, the activity of caspase-9 was significantly increased in the control and treatment groups. We concluded that nanoparticles-loaded Hydroxyurea and it in combination with radiation and hyperthermia induces mitochondrial-dependent apoptosis by down-regulation of caspase-8 and up-regulation of caspase-9 expressions and have higher toxicity effect on MCF-7 cells in comparison with pure Hydroxyurea.

## Introduction

Breast cancer is the most commonly diagnosed cancer among Iranian women (1). A respective disease is the second leading cause of cancer death in women after lung cancer. Chemotherapy is one of the cancer treatment methods in which some certain drugs are employed to destroy cancer cells or to temporarily inhibit cancer. Anti-cancer drugs used in chemotherapy cannot selectively eliminate only cancer cells andcan cause serious damage to nearby healthy tissue. The side effects of chemotherapy depend on the dosage of used drugs (2).

In recent years, extensive researches have been conducted to improve the therapeutic purposes of anticancer drugs. The use of nanotechnology is one of the newest methods in cancer treatment. In this approach, much smaller numbers of anti-cancer drugs arepurposefully carried to the cancerous tissue via nanoparticles. The increase in efficiency of drug and decrease in the complications are some of the important advantages of this treatment method (3-5). Numerous researchers have used nanotechnology for targeted drug delivery to the cancer cells. Gupta* et al. *reported that nanoparticles with a size of 10 to 100 nm are very effective for targeted drug delivery and avoiding reticuloendothelial system (6).

Hydroxyurea (CH_4_N_2_O_2_) is one of the anticancer drugs that are generally used for the treatment of hematologic malignancies, sickle cell anemia, breast cancer, and other diseases (7). Hydroxyurea selectively inhibits ribonucleoside diphosphate reductase, an enzyme required to convert ribonucleoside diphosphates into deoxyribonucleoside diphosphates, thereby preventing cells from leaving the G1/S phase of the cell cycle. Long-term use of high doses of this drug causes adverse reactions in the patients’ blood and skin.Some of the major problems of drug delivery in this method are large amounts of drug release, deposits, and withdrawals of the drug by the reticuloendothelial system (RES). To solve this problem, biodegradable polymers are applied as coatings for nanoparticles containing the drug. Polyethylene glycol (PEG) is one of the most widely used biodegradable polymers to increase stability, efficiency, and the solubility of nanoparticles (8).

Magnetic iron oxide nanoparticles (Fe_3_O_4_ NP) containing Hydroxyurea drug was evaluated in the present study. The nanoparticles using an external magnetic field allowcarrying the drug to the target cells. As well, it is possible to trace these nanoparticles using magnetic the resonance imaging (MRI) (9). In addition, researches have indicated that resonance of superparamagnetic nanoparticles could produce heat under the influence of the external magnetic field. Given that tumor cells are more sensitive to temperature rise compared to the normal cells, this characteristic might be used also to destroy the cancer cells. The programmed cell death (Apoptosis) is a crucial trend required to keep the integrity and homeostasis of multicellular organisms. Interest in programmed cell death or apoptosis was focused originally on its role in tissue homeostasis (10). The importance of apoptosis in the treatment of already established tumors was based on the observation that drugs used in the cancer chemotherapy induce cell death via apoptosis (11). Studies have shown that two main pathways that lead to apoptosis include extrinsic death receptor-dependent and intrinsic mitochondrial-dependent apoptosis. Activation of caspases associated cell deathin both pathways. Caspases 3 is one of the effector caspases, activated via apoptosis initiators. Caspase-8 and Caspase-9 respectively are mainly activated in extrinsic and intrinsic apoptotic pathway (12).

Hyperthermia can encourage apoptosis in normal and tumor cells. The damage caused by hyperthermia is not obvious in normal tissues. In cancerous tissues, the blood vessels have an irregular architecture. The microvascular permeability and blood circulation differ from those of normal tissues as the temperature increases, and damage becomes markedly obvious (13). Moreover, a high temperature can denature the protein, DNA, and RNA damage and interrupt vital cellular processes, ultimately causing cell death. It has been stated that hyperthermia may be a promising treatment for basal cell carcinoma and melanoma by eliciting the intrinsic and extrinsic apoptosis pathways (14). 

It is well known that ionizing irradiation is able to cause cell death. DNA is the critical target, and the radiation generates both single and double-strand DNA breaks. In growing tissues, there is a balance between mitosis and programmed cell death, *i.e.*, apoptosis (15).

Based on the literature review, we decided to investigate the effect of combining Fe_3_O_4_ nanoparticle with Hydroxyurea , radiation, and hyperthermia on the proliferation of human breast adenocarcinoma cell line (MCF-7 cells) and possibleinduction of apoptosis pathway.

## Experimental

*Preparation of nanoparticles containing Hydroxyurea*

In order to prepare nanoparticles containing Hydroxyurea , 1.99 g (0.01 mol) of FeCl_2_.4H_2_O and 5.41 g (0.02 mol) of FeCl_3_.6H_2_O were dissolved in 50 mL of distilled water and polyethylene glycol was added to the solution. Then ammonium hydroxide solution was added drop-by-drop until saturation. The reaction temperature was set on 80 ºC. Then, a homogenizer stirred the solution for 60 min at a speed of 10,000 rpm. After the reaction, the obtained black sediment was washed with distilled water three times and then dried under vacuum. The obtained solution was sonicated using a sonicator (Bandelin Sonorex Digitec) for 5 min to uniformly distribute nanoparticles inside the solution; 15 mg of Hydroxyurea drug was added to 5 mL of suspension of pegylated iron oxide nanoparticles (0.5 mg/mL). Then it was incubated for 48 h at room temperature on a magnetic stirrer at the speed of 600 rpm.

*Analysis of nanoparticle and nanoparticle-loaded Hydroxyurea characteristics*

Size and morphological characteristics of synthesized nanoparticles were assessed using the scanning electron microscope. The surface potential of magnetic iron oxide nanoparticles was assessed by Malvern Zetasizer 3000 HSA. Fourier transform infrared spectroscopy (FTIR) technique was used to ensure the formation of iron oxide nanoparticles bond with the polyethylene glycol polymer and the conversionof polyethylene glycol to acidic polyethylene glycol. Finally, Hydroxyurea loading on pegylated nanoparticles was assessed by FTIR technique.

*Cell culture*

The cells were cultured based on methods described by others (Elengoe and Hamdan 2013). In brief, MCF-7 breast carcinoma cells (ATCC, Manassas, VA) were cultured in DMEM/F12, 10% fetal bovine serum (FBS), 1% penicillin/streptomycin (Gibco, Carlsbad, CA). The Cells were cultured as adherent monolayers (*i.e.*, cultured at approximately 80% confluence) and maintained at 37 °C in a humidified atmosphere of 5% CO2. The cells were harvested with 0.25% trypsin and 1 mM EDTA (Gibco, USA). All chemicals used were of research grade. 

*Study design*

The cultured MCF-7 cells were randomized into T25 flasks and divided into 14 groups. Group 1 was negative control. Group 2 treated with Doxorubicin (800 nM) as the positive control. Group 3-8 treated with the different doses of Hydroxyurea (468.75, 937.5, 1875, 3750, 7500, and 15000 μM). Group 9-14 receiving the different doses of nanoparticle-Loaded Hydroxyurea (468.75, 937.5, 1875, 3750, 7500, and 15000 μM). In the third stage, MCF-7 treated with IC_50_ of Hydroxyurea and nanoparticle-loaded Hydroxyurea in combination with radiation and hyperthermia. After 24 h of incubation, proliferation of MCF-7 cell was evaluated.

*Hyperthermic chemosensitization experiments*

In chemosensitization experiments, the cells were plated in a 96-well plate in the appropriate culture medium. The cells, simultaneously with the drug treatment, were incubated for 1 h at 41 ºC.

*Irradiation*

The cells were irradiated with a single dose from a cobalt-60Co radiation source (gamma radiation). In the experiment, the cells were exposed to a dose of 2Gy with 1.25 MeV.

*Measurement of apoptosis*

Acridine orange (AO) and propidium iodide (PI) were used to visualize living and dead cell for estimating the viability of the cells. The MCF-7 cells were incubated with Hydroxyurea and nanoparticle-loaded Hydroxyurea for 24 h. One group treated with Doxorubicin (800 nM) as positive control. They were then stained with 10 µL/mL acridine orange and incubated for 15 min in 37 °C and Co2 5%. The cells were washed twice with PBS and stained with 10 µL propidium iodide. The cells were then incubated for 5 min in 37 °C and Co2 5%. The cells were again washed with PBS and centrifuged in 1400 rpm for 10 min. The supernatant was removed and 50 µL of the pellet was mounted on the glass slide and observed under fluorescent microscopy. The 100 cells were counted in 5 field and apoptotic and live cells were determined. Acridine orange and propidium iodide are nucleic acid binding dyes that can be used to measure the cell viability. Since acridine orange is cell permeable, all stained nucleated cells generategreen fluorescence. Propidium iodide only enters the cells with compromised membranes and therefore dying, dead, and necrotic nucleated cells stained with propidium iodide generate a red fluorescence.

Acridine orange and propidium iodide are nuclear staining dyes. AO is permeable to both live and dead cells and stains all nucleated cells to generate green fluorescence. PI enters dead cells with compromised membranes and stains all dead nucleated cells to generate red fluorescence (16). 

*Measurement of cell viability*

The cultured cells were harvested, counted (10000 cells per well), and transferred to 96-well plates and incubated for 24 h prior to the addition of appropriate treatments. The treated cells were incubated for 24 h. MTT (3-(4, 5-dimethylthiazol-2-yl)- 2,5-diphenyltetrazolium bromide) (5 mg) was dissolved in 1 mL of phosphate-buffered saline (PBS), and 25 μL of the MTT solution was added to each of the 96-well plate. The plates were wrapped in aluminum foil and incubated at 37 °C for 4 h. The solution in each well, containing media, unbound MTT, and dead cells, was removed by suction, then 200 μL of DMSO was added to each well. The plates were then shaken, and the optical density was measured using a micro plate reader at 492 nm. Three independent experiments were performed for each study and all measurements were performed in triplicate. The results were expressed as the percentage proliferation with respect to vehicle-treated cells. The growth inhibition of at least 50% was considered as cytotoxic. One group was treated with Doxorubicin (800 nM) as the positive control (17).

*Measurement of Caspase-8 and 9 Activities*

A quantitative enzymatic activity assay was carried out according to the instructions of the manufacturer for the colorimetric assay kit. After treatment with various concentrations of Hydroxyurea, nanoparticle-loaded Hydroxyurea and Doxorubicin in 800 nM, cells were washed, collected, lysed, centrifuged, and analyzed for total protein by the Bradford assay. Twenty microlitre of supernatants was added immediately to a buffer containing a p-itroaniline (pNA)-conjugated substrate for caspase-8 (Ac-IETD-pNA) and (LEHD-pNlabeled) for caspase 9. The samples were incubated for 1 h at 37 °C. Absorbance was measured at 405 nm in a plate reader (18).

*Statistical Analysis*

Experimental results were expressed as means ± SD. Statistical analyses were performed using PASW 18.0 (SPSS Inc., Chicago, IL, USA). Model assumptions were evaluated by examining the residual plot. Results were analyzed using a factorial ANOVA with two between-subjects factors. Bonferroni test for pairwise comparisons was used. The differences were considered significant when *P *< 0.05.

## Results

*Characteristics of nanoparticle and nanoparticle-loaded Hydroxyurea*

**Figure 1:**
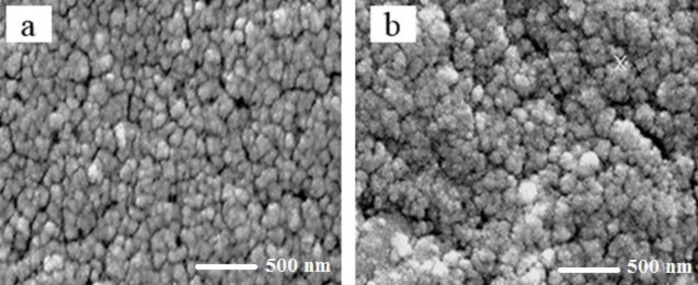
An electron microscope image of the synthesized nanoparticles was used to an examination of the shape and size of them. (a) iron oxide nanoparticles. (b) iron oxide nanoparticles-loaded Hydroxyurea

**Figure 2:**
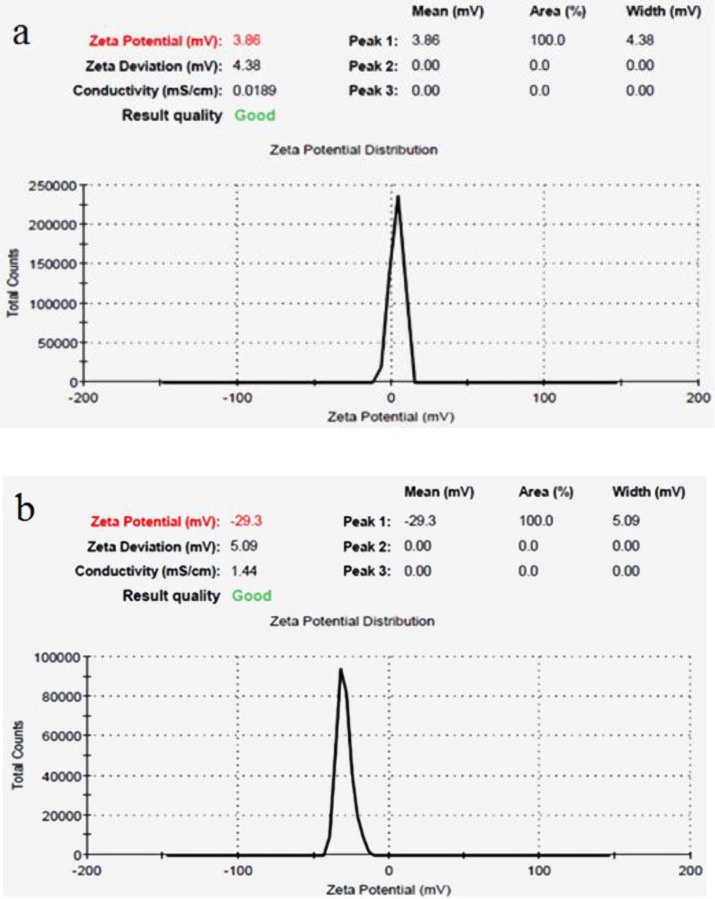
Results of surface potential survey were seen by using Zetasizer to prove the PEGylation of the synthesized nanoparticles. (a) the Surface potential of Iron oxide nanoparticles. (b) PEGylated iron oxide nanoparticles. The change in the surface potential of nanoparticles from 3.86 to -29.3 is due to the negative charge of the PEG carboxyl group

The mean size of magnetic iron oxide nanoparticles was obtained 26 nm using scanning electron microscopy (Figure 1a). In addition, the mean size of iron oxide nanoparticles containing Hydroxyurea was determined 48 nm (Figure 1b). In both samples, the nanoparticles were observed in a spherical shape with smooth and uniform surfaces (Figures 1a and 1b). The surface potential of nanoparticles provides valuable information about their size and surface charge. The surface charge of nanoparticles is one of the important factors of stability in solutions. In this study, according to the results of Zetasizer technique, the surface potential of magnetic iron oxide nanoparticles was obtained 3.86 mV (Figure 2a) and the surface potential of pegylated magnetic iron oxide nanoparticles was -29.3 mV (Figure 2b). Negative surface potential is caused by a negative charge of carboxyl groups of the PEG.

**Figure 3:**
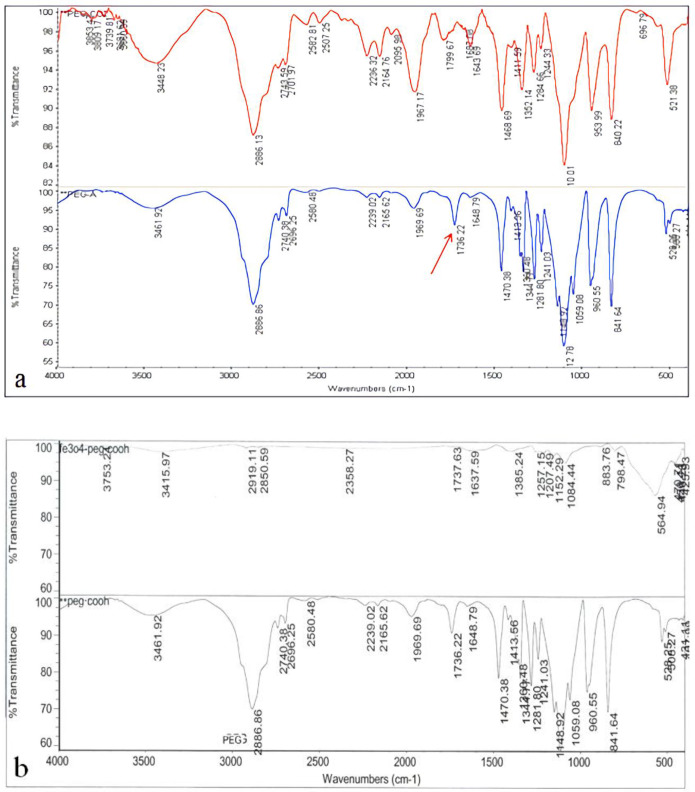
In order to investigate the formation of acidic polyethylene glycol and Nanoparticles PEGylation, the FTIR spectrometry was used. (a) the formulation of acidic polyethylene glycol; the arrow represents the specific bond of the polyethylene glycol acid. (b) PEGylation of iron oxide nanoparticles with acidic polyethylene glycol

One of the important steps in the PEGylation of nanoparticles is the production of acidic polyethylene glycol. As seen in Figure 3a, the results of the FTIR analysis indicate that acidic polyethylene has been formed. The arrow represents the bond of the acidic polyethylene glycol. The success of the PEGylation of Iron Oxide Nanoparticles was also evaluated by FTIR method. The results showed that the synthesized nanoparticles were well PEGylated by acidicpolyethylene glycol (Figure 3b).

*Apoptosis Result*

**Figure 4:**
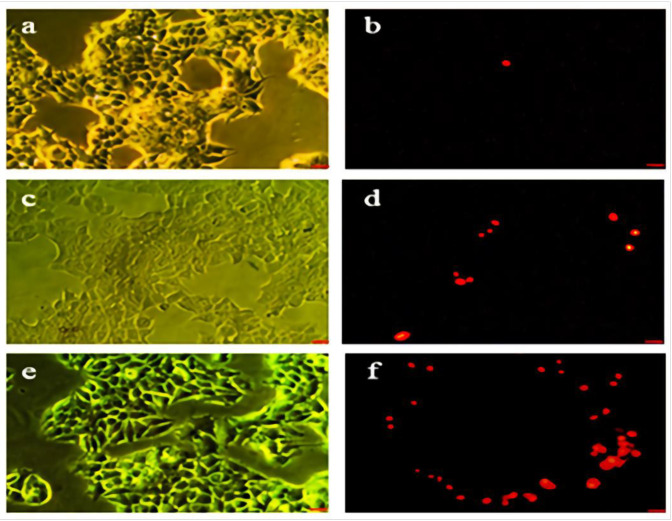
Phase contrast and fluorescent images of treated and non treated MCF-7 cell line. (a and b) the control group, (c and d) cells which pure Hydroxyurea was added in their culture medium. (e and f) cells which nanoparticles-loaded Hydroxyurea was added in their culture medium. Scale bar = 100 µm

As shown in Figures 4a-4f the green cells (excluding propidium iodide) are viable with diffused chromatin, whereas the cells with condensed chromatin are apoptotic. Further, the red cells (including propidium iodide) with non-condensed chromatin are necrotic. The apoptosis percent determination with propidium iodide staining using various concentrations of Hydroxyurea (Figures 4c and 4d) and nanoparticle-loaded Hydroxyurea (Figures 4e and 4f) in treatment groups within 24 h. The nanoparticle-loaded Hydroxyurea treated groups had the highest and the Hydroxyurea groups had the lowest apoptosis percent (*P *< 0.05). Interestingly that apoptosis percent increased with increasing the concentration of nanoparticle-loaded Hydroxyurea in compared with Hydroxyurea .

*Cell viability Result*

**Figure 5:**
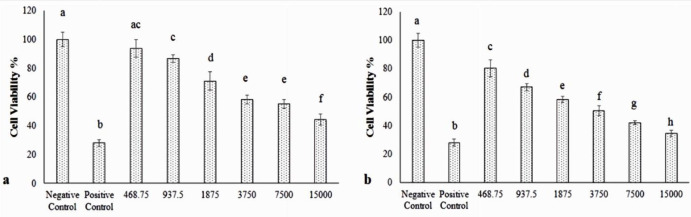
MTT assay results of different doses of (a) pure Hydroxyurea and (b) nanoparticle-loaded Hydroxyurea on cell viability of MCF-7 cells. Different letters above the columns show statistically significant differences between the groups (*P *< 0.05).

MTT assay findings in various concentrations of Hydroxyurea (Figure 5a) and nanoparticle-loaded Hydroxyurea (Figure 5b) in treatment groups within 48 h. The nanoparticle-loaded Hydroxyurea treated groups had the highest and the Hydroxyurea groups had the lowest cytotoxicity (*P *< 0.05). Interestingly that cytotoxicity increased with increasing the concentration of nanoparticle-loaded Hydroxyurea in compared with Hydroxyurea. MTT technique was used to determine IC_50_ and the results were 9814 µM for Hydroxyurea drug and 2635 µM for nanoparticles containing Hydroxyurea.

*Cell viability and Apoptosis Result in thermoradiotherapy groups*

**Figure 6:**
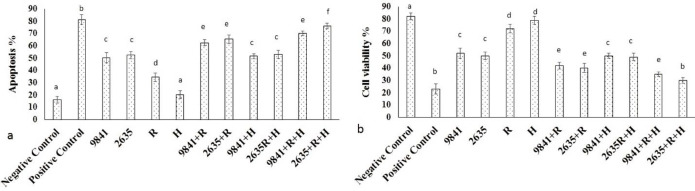
The MTT assay results. (a) appoptosis and (b) cell viability of IC_50_ of pure Hydroxyurea (9841), nanoparticle-loaded Hydroxyurea (2635), Radiation (R), Hyperthermia (H) and multi agenttreatment groups on MCF-7 cells. Different superscript letters show statistically significant differences between the groups (*P *< 0.05).

MTT assay findings in IC_50_ of Hydroxyurea (Figure 6a) and nanoparticle-loaded Hydrox-yurea and thermoradiotherapy group within 48 h. The nanoparticle-loaded Hydrox-yurea with thermoradiotherapy treated groups had the highest and the hyperthermia group had the lowest cytotoxicity (*P *< 0.05). Apoptosis findings in IC_50_ of Hydroxyurea (Figure 6b) and nanoparticle-loaded Hydroxyurea and thermoradiotherapy group within 48 h. The nanoparticle-loaded Hydroxyurea with thermoradiotherapy treated groups had the highest and the hyperthermia group had the lowest apoptosis percent (*P *< 0.05).

*Measurement of caspase-8 and -9 activities*

To explore the possible biochemical mechanisms underlying treatment-induced apoptosis, the activation of caspase-8 and -9 were assayed. The results demonstrated that the activity of caspase-8 did not show significant changes. However, the activity of caspase-9 was significantly increased in the control and treatment groups. The results confirmed that Hydroxyurea and nanoparticle-loaded Hydroxyurea induced mitochondrial-dependent apoptosis by down-regulation and up-regulation of expressions of caspase-8 of caspase-9, respectively (Table 1).

## Discussion

Despite increasing the chemotherapeutic drugs, extensive side effects in chemotherapy is one of the main problems in the use of anticancer drugs. Targeted drug delivery has attracted more attention by researchers in order to reduce these complications. Drug delivery is divided into two passive and active targeting methods (19). In passive delivery, some of the tumoral tissue characteristics such as permeable endothelium *etc.* are used for drug delivery. Inactive type, the specific characteristics of tumoral cells are applied for better drug delivery.

 One of the common techniques in the active targeting method is polydrug conjugates. Various strategies can be employed in this approach. One of these strategies is to conjugate the drug directly to the ligand of the target cell. The fundamental problem of this strategy is the reduction of anti-tumor power of the drug due to the inactivation of the cell-binding domain (20, 21). Another strategy is the use of polymers as themediator for the binding of drugs to the cells. For example, in previous studies, the anticancer drug of Doxorubicin in combined with HPMA polymer was used to treat cancer (22).

In other studies, polyethylene glycol polymer has also been used. For example, in a study, PEG-conjugated Doxorubicin drug was used for the treatment of melanoma and leukemia. The *in-vivo *pegylation of nanoparticles not only increases plasma half-life but also prevents opsonization. In addition, the size of the PEGylated particle was increased, so lower amount was excreted via the kidneys. Although this combination has increased the cell death rate compared to the control group, the toxicity level has been lower than the pure drug (23).

 In our study, acidic polyethylene glycol was used to pegylation of nanoparticles. Combination of PEG with Hydroxyurea drug increased its toxicity compared to the pure drug.A study conducted on the effect of PEG in increasing the efficiency of drug delivery indicates that the use of PEG combined with anti-cancer drugs such as Ara-c and Melphalan increases the toxicity of the drug (24). The results of this study are in line with our results.

Inour study, magnetic Fe_3_O_4_ nanoparticles were used for drug delivery. Magnetic iron oxide nanoparticles are one of the best options for targeted drug delivery in the treatment of cancer due to its long half-life in the blood circulation, biodegradability, and low toxicity. The extensive surface of nanoparticles could cause the formation of multiple active groups to bind with specific ligands. The advantage of using these nanoparticles compared to other nanoparticles is that these ones or cells containing nanoparticles could be tracked using MRI imaging after injection into the body. For example, Remsen *et al. *utilized magnetic iron oxide nanoparticles conjugated to the specific tumoral antibody for imaging of brain tumors in rats (25).

In the present study, toxic responses of various concentrations of nanoparticle-loaded Hydroxyurea and Hydroxyurea to human breast epithelial MCF-7 cells were investigated. Our findings demonstrated that exposure of Hydroxyurea to MCF-7 cells in combination with Fe_3_O_4_ nanoparticles caused cytotoxicity. We also observed the apoptotic response of nanoparticle-loaded Hydroxyurea to MCF-7 cells in compared Hydroxyurea groups significantly increased with increasing the concentration of nanoparticle-loaded Hydroxyurea . IC_50_ level was obtained 9814 µM for Hydroxyurea drug and 2635 µM for nanoparticle containing Hydroxyurea . This significant decline in the IC_50_ level indicates that the use of pegylated nanoparticle-loaded Hydroxyurea would be more efficient than using a pure drug. 

Apoptosis is a process that can be initiated by a number of different stimuli. Drug and nanoparticle have been considered to be one such initiator (26). The degree of induction, however, has been controversial, and no defined doses or dose rates have been proposed to frame the therapeutic window that can be employed for therapy, especially radiochemotherapy (27). Activation of caspases is generally considered to be a requisite event during apoptosis. Our results also demonstrated that apoptosis induced by nanoparticle-loaded Hydroxyurea was dependent on the activation of caspase-9, suggesting a possible mitochondrial apoptotic pathway, *e.g.* through calcium-dependent cytochrome c release or a change in Bax/Bcl-2 ratio. These findings were in agreement with other studies obtained in the cladribine-treated cell line (28). The present study revealed that nanoparticle-loaded Hydroxyurea induced mitochondrial-dependent apoptosis by down-regulation of expression caspase-8 and up-regulation of expression caspase-9.

In the present study, toxic responses of irradiation, hyperthermia, and IC_50_ of Hydroxyurea and nanoparticle-loaded Hydroxyurea , as well as their combination in different groups to human breast epithelial MCF-7 cells, were investigated. Our findings demonstrated that exposure of nanoparticle-loaded Hydroxyurea to MCF-7 cells in combination with hyperthermia and irradiation caused cytotoxicity. We also observed the apoptotic response of various combinations to MCF-7 cells with hyperthermia and irradiation. MTT assays revealed that nanoparticle-loaded Hydroxyurea with radiation treatment showed the highest cytotoxicity among the other treatments.

**Table 1:** Activity of caspase 8 and -9 (Mean ± SD) in the MCF-7 cells after treatment

**Groups**	**Mean ± SD of Variables**			
	** Hydroxyurea **	** Hydroxyurea - loaded nanoparticles**	** Hydroxyurea **	** Hydroxyurea - loaded nanoparticles**
	**Caspase-8 activity**	**Caspase-8 activity**	**Caspase-9 activity**	**Caspase-9 activity**
Negative Control	0.171 ± 0.04	0.171 ± 0.04	0.145 ± 0.02	0.145 ± 0.02
Positive Control	0.239 ± 0.06	0.239 ± 0.06	0.546 ± 0.036	0.546 ± 0.036
468.75	0. 216 ± 0.063	0.143 ± 0.056	0.2 ± 0.014	0.2 ± 0.014
937.5	0.218 ± 0.037	0.185 ± 0.029	0.21 ± 0.023	0.256 ± 0.023
1875	0.219 ± 0.064	0.195 ± 0.052	0.256 ± 0.029	0.301 ± 0.016
3750	0.228 ± 0.05	0.208 ± 0.042	0.289 ± 0.025	0.356 ± 0.02
7500	0.243 ± 0.04	0.213 ± 0.036	0.356 ± 0.03	0.398 ± 0.011
15000	0.247 ± 0.04	0.235 ± 0.02	0.4 ± 0.041	0.456 ± 0.026
*P*-values	*P *> 0.05	*P *> 0.05	*P *< 0.05	*P *< 0.05

## Conclusion

The results obtained from this study showed that the IC_50_ level of nanoparticle-loaded Hydroxyurea in combination radiation and hyperthermia on MCF-7 cell line was significantly less than pure Hydroxyurea after 48 h. Also, we concluded that Hydroxyurea in combination with Fe_3_O_4_ nanoparticles induces mitochondrial-dependent apoptosis by down-regulation of caspase-8 and up-regulation of caspase-9 expressions. The understanding of the mechanisms involved and synergistic effects of mentioned therapy regimens and order of their application in cancer therapy process to achieve an effective modality needs further studies.
